# Deficiency of CCAAT/enhancer binding protein family DNA binding prevents malignant conversion of adenoma to carcinoma in NNK-induced lung carcinogenesis in the mouse

**DOI:** 10.1186/1476-4598-11-90

**Published:** 2012-12-12

**Authors:** Shioko Kimura, Jorge Paiz, Mitsuhiro Yoneda, Taketomo Kido, Charles Vinson, Jerrold M Ward

**Affiliations:** 1 Laboratory of Metabolism, National Cancer Institute, National Institutes of Health, Bethesda, Maryland, 20892, USA; 2 Global VetPathology, Montgomery Village, Maryland, 20866, USA

**Keywords:** C/EBPs, Lung chemical carcinogenesis bioassay, Dominant negative, A-C/EBP, Transgenic mouse, 4-(methylnitrosamino)-1-(3-pyridyl)-1-butanone, NNK

## Abstract

**Background:**

The CCAAT/enhancer binding proteins (C/EBPs) play important roles in carcinogenesis of many tumors including the lung. Since multiple C/EBPs are expressed in lung, the combinatorial expression of these C/EBPs on lung carcinogenesis is not known.

**Methods:**

A transgenic mouse line expressing a dominant negative A-C/EBP under the promoter of lung epithelial Clara cell secretory protein (*CCSP*) gene in doxycycline dependent fashion was subjected to 4-(methylnitrosamino)-1-(3-pyridyl)-1-butanone (NNK)-induced lung carcinogenesis bioassay in the presence and absence of doxycycline, and the effect of abolition of DNA binding activities of C/EBPs on lung carcinogenesis was examined.

**Results:**

A-C/EBP expression was found not to interfere with tumor development; however, it suppressed the malignant conversion of adenoma to carcinoma during NNK-induced lung carcinogenesis. The results suggested that Ki67 may be used as a marker for lung carcinomas in mouse.

**Conclusions:**

The DNA binding of C/EBP family members can be used as a potential molecular target for lung cancer therapy.

## Background

The CCAAT/enhancer binding proteins (C/EBPs) are a family of basic leucine zipper (B-ZIP) transcription factors that play important roles in cellular differentiation, proliferation, survival, and apoptosis, and metabolism, inflammation, and transformation [1-3]. Six C/EBP family members have been identified that share the N-terminal basic amino acid-containing region necessary for DNA binding and the highly conservative C-terminal leucine zipper (B-ZIP) dimerization motif [4,5]. Through the B-ZIP domain, they can homo- and/or hetero-dimerize with each other to bind specific DNA sequences [4,5].

Among the family members, the most studied are C/EBPα and C/EBPβ. C/EBPα is responsible for blocking proliferation, promoting differentiation and suppressing tumorigenesis, thus being considered as a tumor suppressor. Significant down-regulation of C/EBPα expression is found in cancers of various tissues such as mammary gland [6], lung [7], and the epidermis [8]. Further, specific somatic mutations are found in ~10% of acute myeloid leukemia (AML) patients [9,10]. The reintroduction of C/EBPα blocked the *in vivo* tumorigenicity of AML and skin carcinogenesis [11,12]. In contrast, C/EBPβ is more complex, but has been implicated in playing a role in tumorigenesis, depending on the cellular context and C/EBPβ forms present, out of three translational isoforms [3]. In particular, one of the C/EBPβ isoforms, the C/EBP-liver-enriched inhibitory protein acts as dominant-negative, and the increased expression inhibits the transcriptional activation of genes involved in differentiation, while its over-expression is found in breast cancers [13,14].

In the lung, three C/EBPs, C/EBPα, C/EBPβ, and C/EBPδ, are highly expressed with various degrees of expression depending on protein subtypes, cell types, and/or developmental stages [15-18]. Similar to tumors of other organs, C/EBPα is described as a tumor suppressor in lung cancer [7,19], while C/EBPβ regulates the expression of Matrix Metalloproteinase (MMP) 1 that mediates extracellular matrix remodeling and promotes tumor invasion [20]. The role of C/EBPδ in lung carcinogenesis is not clear. Because of multiple forms of C/EBPs expressed in the lung, how C/EBPs as a whole contribute to lung carcinogenesis is not known.

A-C/EBP is a dominant negative form of C/EBP that contains leucine zipper dimerization domain and an acidic region, replacing the basic region of C/EBPs. A-C/EBP heterodimerizes with all C/EBP family members through the leucine zipper region of the B-ZIP, and the acidic region forms coiled coil structure to stabilize the heterodimer, thus blocking the DNA binding of C/EBPs [4]. A transgenic mouse line (*TetO-A-C/EBP*) was established that expresses the A-C/EBP dominant negative gene [4] under the regulated control of the tetracycline operon (TetO) promoter [21]. The *TetO-A-C/EBP* mice were further crossed with transgenic mice *CCSP-rtTA* that express the rtTA under the promoter of lung epithelial Clara cell-specific *CCSP* (*SCGB1A1*) gene [18]. The progeny of this cross, named *CCSP-rtTA;TetO-A-C/EBP* mice were previously shown to highly express A-C/EBP in the presence of doxycycline and suppress the transcription of C/EBP-responsive genes such as secretoglobin 3a2 in lung [18,22].

In this study, *CCSP-rtTA;TetO-A-C/EBP* mice were subjected to 4-(methylnitrosamino)-1-(3-pyridyl)-1-butanone (NNK)-induced lung carcinogenesis bioassay [23] in the presence and absence of doxycycline, and the effect of A-C/EBP expression on the development and/or progression of lung cancer was examined.

## Results

**Figure 1:**
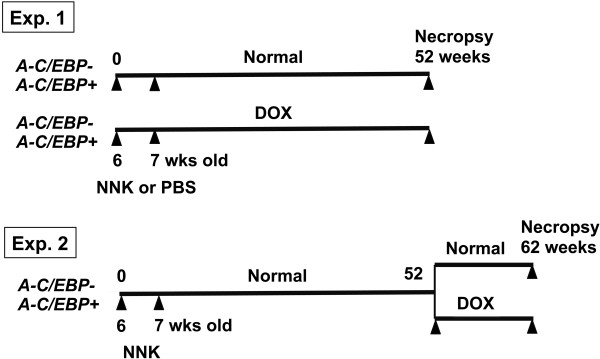
**NNK carcinogenesis bioassay schemes.** In Experiment 1, *A-C/EBP*+ and – mice were intraperitoneally administered NNK or PBS at 6 and 7 weeks of age, a half of which were kept under normal diet while the other half under DOX diet until necropsy that was carried out at 52 weeks post 1^st^ NNK administration. In Experiment 2, *A-C/EBP*+ and – mice were intraperitoneally administered NNK at 6 and 7 weeks of age. They were maintained under regular diet for 52 weeks after 1^st^ NNK administration; half remained under regular diet while the other received DOX diet for additional 10 weeks. They were subjected to necropsy at 62 weeks post 1^st^ NNK administration.

**Figure 2:**
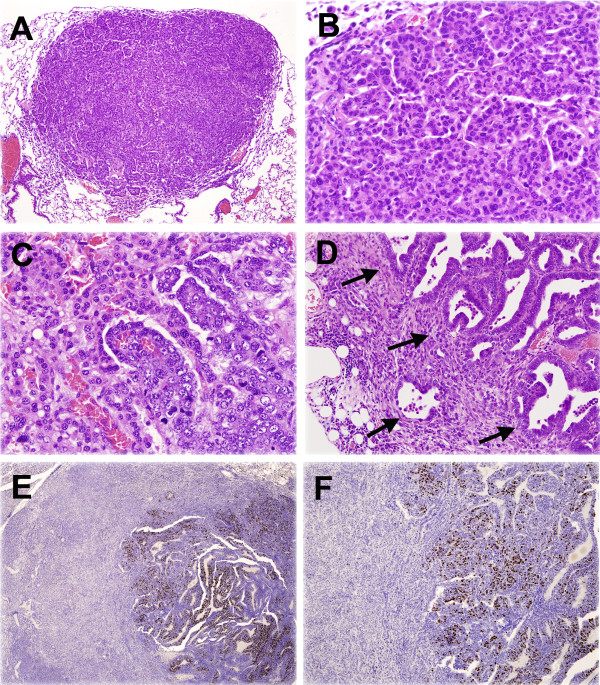
**Representative histology of NNK-induced lung tumors in mice**. (**A**) Alveolar adenoma at low magnification. (**B**) High magnification of alveolar adenoma shown in A. (**C**) Carcinoma (middle and right side) in adenoma (left side of figure). (**D**) Invasive adenocarcinoma into pleura and mediastinal fat (invasive front shown by arrows). (**E**, **F**) Adenocarcinoma in adenoma for Ki67 staining. Only adenocarcinoma is highly positive for Ki67. Original magnification: A, 100X, B, C, 400X, D, F, 200X, E, 40X.

The mean body weights of mice fed Dox-containing diet when measured at 52 weeks 1^st^ post-NNK administration were in general larger than those of respective group of mice fed regular diet in both genotypes (Figure 1, Table 1). This was particularly evident in NNK-treated group of mice with statistically significant difference (group 1 vs. 5 and 2 vs. 6, P<0.00001). As expected, the NNK-treated group of mice developed higher number of alveolar hyperplasias, adenomas, and/or adenomas and carcinomas per mouse with statistical significance as compared to their respective saline-treated group, regardless of Dox administration (Figure 2A, B and Table 1, group 1 vs. 3, 2 vs. 4, 5 vs. 7, and 6 vs. 8). Only a few of them developed carcinomas. There was a slight significant increase in the incidence of alveolar hyperplasia with NNK-treated, regular-diet group of mice as compared with their respective Dox diet group (group 1 vs. 5). However, no significant differences were found in the numbers of these lesions between *A-C/EBP+* and *A-C/EBP-* mice. Only slight decreases in tumor incidence with almost no statistical significance (P=0.0497) was noted in NNK-treated, regular diet *A-C/EBP-* mice relative to those of *A-C/EBP+* mice (group 5 vs. 6).

**Table 1:** **Tumor multiplicities/incidences in NNK or saline-treated *A-C/EBP- or A-C/EBP+ *mice fed Dox or regular diet and analyzed at 52 weeks post-NNK/saline administration**

**Group**	**Genotype (*A-C/EBP***	**Treatment**	**Diet**	**No. of mice**	**Body weight**	**Gross tumor**		**Histological**	**lesions**		**Tumor incidence**
	**+ or -**^**a**^**)**			**examined**	**(g)**	**per mouse**	**Alveolar hyperplasia per mouse**	**Adenoma per mouse**	**Carcinoma per mouse**	**Adenoma and carcinoma per mouse**	**(%)**^**b**^
1	-	NNK	Dox	45	46.2±0.8^d,e^	1.00	0.11^g^	1.20^h^	0.044	1.20^h^	76 (34)^c^
2	+	NNK	Dox	30	43.7±0.8^e^	1.20	0.23	1.30^h^	0.13	1.40^h^	83 (25)
3	-	Saline	Dox	24	43.7±1.1	0.29	0.08	0.21	0	0.21	21 (5)
4	+	Saline	Dox	23	43.4±0.8^f^	0.26	0.17	0.17	0	0.17	13 (3)
5	-	NNK	Regular	44	39.8±0.8	1.00	0.36	1.30^h^	0	1.30^h^	71 (31)^i^
6	+	NNK	Regular	40	38.4±0.9	1.00	0.30	1.50^h^	0.025	1.60^h^	88 (35)
7	-	Saline	Regular	38	40.8±1.2	0.27	0.18	0.32	0	0.32	26 (10)
8	+	Saline	Regular	31	39.9±0.9	0.26	0.19	0.26	0	0.26	19 (6)

^a^*A-C/EBP*- is considered as wild-type control.

^b^Percentage of mice with adenomas or carcinomas.

^c^Numbers in parentheses show the number of mice having tumors.

^d^Significantly different from their respective *A-C/EBP+* group (group 1 vs. 2) at P<0.05 by student *t*-test.

^e^Significantly different from their respective regular diet group (group 1 vs. 5, and 2 vs. 6) at P<0.00001 by student *t*-test.

^f^Significantly different from their respective regular diet group (group 4 vs. 8) at P<0.01 by student *t*-test.

^g^Significantly different from their respective regular diet group (group 1 vs. 5) at P<0.05 by student *t*-test.

^h^Significantly different from their respective saline group (group 1 vs. 3, 2 vs. 4, 5 vs. 7, and 6 vs. 8) at P<0.0001 by student *t*-test.

^i^Significantly different from their respective *A-C/EBP+* group (group 5 vs. 6) at P=0.0497 by Fisher’s exact test.

In order to confirm that A-C/EBP was indeed expressed in *A-C/EBP+* mice, lung total RNAs were isolated from several mice at 52 weeks post-NNK for qRT-PCR analysis. The *A-C/EBP+* mice kept under Dox diet had 2–10 x 10^6^ fold higher A-C/EBP expression as compared to no expression of mice with regular diet (data not shown, Ct value 14–17 vs. 34–35, respectively). These results demonstrated that the expression of A-C/EBP in *A-C/EBP+* mice did not have any effect on NNK-induced lung tumor multiplicity or incidence.

The effect of A-C/EBP expression on the NNK-induced adenomas/carcinomas was examined in the second set of experiment (Figure 1). For this, both *A-C/EBP-* and *A-C/EBP+* mice were NNK-treated and maintained with a regular diet for 52 weeks. Thus, they corresponded to group 5 and 6 mice shown in Table 1, respectively. At 52 weeks post-1^st^ NNK treatment, a half of them started being fed Dox-containing food, while the other half were kept under regular diet for additional 10 weeks. They were subjected to lung necropsy at 62 weeks post-1^st^ NNK administration. Similar to the 52 weeks mice, Dox administered mice were larger in weights as compared to regular diet mice (Table 2, group 1, 2 vs. 3, 4). There were no differences in the numbers of gross tumors, alveolar hyperplasias, adenomas, and adenomas and carcinomas combined between *A-C/EBP+* and *A-C/EBP-* mice with and without Dox diet. However, interestingly, the carcinoma numbers per mouse were significantly lower in *A-C/EBP+* mice as compared with *A-C/EBP-* mice when they were fed Dox-containing diet for 10 weeks from 52 weeks post-1^st^ NNK administration (group 1 vs. 2) as determined by histopathological analysis. The carcinoma numbers in *A-C/EBP+* mice were also significantly lower as compared to their respective regular diet group (group 2 vs. 4). In 52 weeks NNK-treated mouse lungs, there were almost no carcinomas found regardless of genotype or diet (compare the number of carcinomas between Table 1 and 2), suggesting that the presumable malignant conversion from adenoma to carcinoma took place between 52 and 62 weeks. In fact, some carcinomas were found inside adenomas, suggestive of this transition (Figure 2C). Malignant tumors were mostly papillary (Figure 2D).

**Table 2:** **Tumor multiplicities/incidences in NNK-treated *A-C/EBP- or A-C/EBP+ *mice fed Dox or regular diet at 52 weeks post-NNK administration for additional 10 weeks and analyzed at 62 weeks post-NNK administration**

**Group**	**Genotype (*A-C/EBP***	**Treatment**	**Diet during **	**No. of mice**	**Body weight**	**Gross tumor**		**Histological**	**lesions**		**Tumor incidence**
	**+ or -**^**a**^**)**		**52-62 weeks**	**examined**	**(g)**	**per mouse**	**Alveolar hyperplasia per mouse**	**Adenoma per mouse**	**Carcinoma per mouse**	**Adenoma and carcinoma per mouse**	**(%)**^**b**^
1	-	NNK	Dox	19	42.2±1.4^d^	2.00	0.37	1.63	0.37^e^	2.00	95 (17)^**c**^
2	+	NNK	Dox	18	44.5±1.5^d^	2.83	0.33	2.06	0.056^f^	2.11	83 (15)
3	-	NNK	Regular	18	36.2±1.6	1.89	0.11	1.50	0.44	1.94	94 (16)
4	+	NNK	Regular	18	38.1±1.6	2.22	0.17	2.00	0.44	2.44	78 (14)

^a^*A-C/EBP-* is considered as wild-type control.

^b^Percentage of mice with adenomas or carcinomas.

^c^Numbers in parentheses show the number of mice having tumors.

^d^Significantly different from their respective regular diet group (group 1 vs. 3, 2 vs. 4) at P<0.01 by student *t*-test.

^e^Significantly different from their respective *A-C/EBP+* group (group 1 vs. 2) at P<0.05 by student *t*-test.

^f^Significantly different from their respective regular diet group (group 2 vs. 4) at P<0.05 by student *t*-test.

In order to further obtain insight into the malignant conversion of adenoma to carcinoma, immunohistochemistry for various markers were carried out using lung sections prepared at 62 weeks (Table 2). Antibodies used included those for vascular endothelial growth factor (VEGF) as a marker for angiogenesis, Ki67 as a marker for proliferation, and MMP 2 and 9 that are known to be highly expressed in cancerous tissues and play a role in their invasion, metastasis and angiogenesis [24,25]. TUNEL assay was also carried out to detect apoptotic cells. The results demonstrated that no clear positive staining was obtained for VEGF and TUNEL in either adenomas or carcinomas, while MMP 2 and 9 were positive for both adenomas and carcinomas (data not shown). In contrast, Ki67 was highly positive for carcinomas, clearly demarcating the adenomas in the majority of lungs that were histopathologically diagnosed as those having carcinomas (Figure 2E, F). Only one section showed higher positive staining in benign areas than carcinomas (1 out of 73 lung sections analyzed). Together, these results suggested that A-C/EBP expression in the presence of Dox may interfere with malignant conversion of adenoma to carcinoma in *A-C/EBP*+ mice.

## Discussion

The current study using transgenic mice expressing dominant negative A-C/EBP in lung in Dox-inducible fashion, demonstrated that the expression of A-C/EBP did not have any effect on the development of lung adenomas in NNK-induced lung carcinogenesis bioassay. Interestingly, however, A-C/EBP expression appeared to have inhibited the malignant conversion of adenoma to carcinoma in mice expressing A-C/EBP when the expression was initiated at 52 weeks post-1^st^ NNK administration, the time when tumors (mostly adenomas) had already been well developed (see Table 1), and the mice necropsied at 62 weeks post-1^st^ NNK administration. Malignant progression of mouse lung adenomas to carcinomas has been postulated [26,27].

In the current study, the evaluation of malignant phenotypes was mainly carried out by histopathological analysis. The diagnosis of the tumors was based on nomenclature published by the international veterinary pathology committees [28]. Malignant mouse lung tumors clearly showed a malignant morphologic pattern. While there are no published standards for the diagnosis of malignant lung tumors of mice using immunohistochemistry, our results suggest that Ki67 may be used as a marker for carcinomas in lung carcinogenesis in mice. The correlation of Ki67 with malignant phenotypes has been reported for human cancers [29-31].

Previously, the effect of A-C/EBP expression on skin carcinogenesis was examined using *K5tTA;TetO-A-C/EBP* mice, which expressed A-C/EBP in their epidermis under keratin 5 (K5) promoter in the absence of Dox [21]. When A-C/EBP expression was initiated by giving the mice regular diet at weaning age, followed by subject to a skin carcinogenesis assay at 8 weeks of age, they developed reduced number of squamous papillomas. Further, tumor regression was observed when *K5tTA;TetO-A-C/EBP* mice were first allowed to develop skin papillomas in the presence of Dox, and then Dox was withdrawn from the diet [21]. Most papillomas regressed within 4 weeks of the start of Dox diet. Thus the effect of A-C/EBP on skin papilloma development appeared different from that of NNK-induced lung carcinogenesis found in this study. The phenomenon found in the skin carcinogenesis study was due to A-C/EBP-induced expression of p53 and apoptosis, partially acting through C/EBPβ [21].

Lung expresses three C/EBPs, C/EBPα, C/EBPβ, and C/EBPδ at various degrees depending on protein subtypes, cell types, and/or developmental stages [15-18]. How these multiple C/EBPs interact and/or interfere with each other to regulate downstream genes, particularly those involved in carcinogenesis is not known. C/EBPα is well established as a tumor suppressor in many tumors including lung [7,19], while C/EBPβ is suggested to be involved in lung tumorigenesis [3,13,14]. Although the role of C/EBPδ in lung carcinogenesis has not been known, it is generally considered as tumor suppressor. However, it may also play a role in tumor progression and metastasis [32,33]. Based on the opposite and/or dual roles that each C/EBP plays, lung carcinogenesis may be influenced by a slight drift from the delicate balance built among activities of these C/EBPs. It is possible that the expression of A-C/EBP might affect malignant conversion of lung tumors through C/EBPβ and/or C/EBP δ. The precise mechanism for the effect of A-C/EBP on NNK-induced lung carcinogenesis, and how that explains the difference between the previous skin carcinogenesis and the current lung carcinogenesis studies require further experiments.

In the current carcinogenesis study, we found that Dox did not have any effect on NNK-induced lung carcinogenesis, even though Dox was demonstrated to inhibit tumor growth and tumor-associated vascular hyperpermeability [34]. Only difference found in the current study was that Dox-treated groups of mice had slight weight gain as compared to regular diet groups. We do not know the reasons for this phenomenon.

In our previous study, the expression levels of A-C/EBP in *A-C/EBP+* mice were already elevated approximately 3 fold after 3 days of Dox exposure, which were further increased to 10 fold in lungs of 4 months-old *A-C/EBP+* mice as compared to *A-C/EBP-* control mice [22]. In the current study, *A-C/EBP+* mice maintained under Dox feed for more than a year exhibited 2–10 × 10^6^ fold higher A-C/EBP expression as compared to no expression of mice with regular diet. These results demonstrated that *A-C/EBP+* mice indeed expressed A-C/EBP throughout their lives under Dox feed, and the expression level appeared kept increasing. With this level of A-C/EBP, it can be said that all DNA binding activities of C/EBPs were likely to be totally abolished during the entire period of carcinogenesis study.

## Conclusions

The total abolition of DNA binding activity of C/EBPs through A-C/EBP expression may not contribute to the development of adenomas, but may play a role in the malignant conversion from adenomas to carcinomas during NNK-induced lung carcinogenesis in mice. The results implicate that the DNA binding of C/EBP family members can be used as a potential molecular target for the therapy of lung cancers. The results further suggest that Ki67 may be used as a marker for lung carcinogenesis in mice.

## Methods

### Animals and chemicals

The mice used in this study were *CCSP-rtTA;TetO-A-C/EBP* and *CCSP-rtTA* mice. The details for characterization and genotyping were previously described [18,22]. Mice carrying *A-C/EBP* gene (*CCSP-rtTA;TetO-A-C/EBP*) were tentatively termed *A-C/EBP+* while mice not carrying *A-C/EBP* gene (*CCSP-rtTA*) were termed *A-C/EBP*-. The *A-C/EBP+* mice expressed A-C/EBP in the presence of doxycycline while the *A-C/EBP*- mice did not. The latter was considered as the wild-type control. 4-(Methylnitrosamino)-1-(3-pyridyl)-1-butanone (NNK) was purchased from Toronto Research Chemicals, Inc. (North York, Ontario, Canada). Mice for these experiments were bred in our animal facility.

### Animal treatment

Male mice were used for all experiments. For the 1^st^ set of experiment, *A-C/EBP+* and *A-C/EBP-* mice were intraperitoneally injected with NNK (dissolved in saline, 103 mg/kg per injection) or saline as control at 6 and 7 weeks of age (Figure 1). A half of the NNK or control group of mice were fed food containing 200 mg/kg doxycycline (Dox), and the other half fed control diet from birth continuously until necropsy, at 52 weeks post-1^st^ NNK administration. For the 2^nd^ experiment, all mice were injected with NNK as described above and were maintained under normal feed for 52 weeks. At 52 weeks post-1^st^ NNK treatment, half of them were fed Dox-containing food while the others stayed with the regular diet for an additional 10 weeks. They were killed at 62 weeks post-1^st^ NNK administration. All experiments were conducted in accordance with the National Institutes of Health guidelines after approval by the National Cancer Institute (NCI) Animal Care and Use Committee.

### Lung tumor analysis

Gross number of lung tumors was counted at the time of necropsy. Lungs were fixed in 10% neutral buffered formalin, embedded in paraffin, sectioned at 5 μm thickness, and stained with hematoxylin and eosin for histological examination. The number of lung nodules was counted grossly and histologically, and the number of alveolar hyperplasias, adenomas, and carcinomas were recorded per mouse using a whole lung section [28,35]. The statistical analysis was carried out using student *t*-test for tumor multiplicity and Fisher’s exact test for tumor incidence. P<0.05 was considered as statistical significant.

### Immunohistochemistry

Immunohistochemical staining was carried out by the avidin-biotin-peroxidase complex method (Vector Laboratories, Burlingame, CA) using rabbit anti-Ki67 antibody (1:500, Abcam, Cambridge, MA). Immunostaining was visualized using 3, 3’-diaminobenzidine (DAB) as substrate (DAKO, Carpinteria, CA) and counterstained with hematoxylin (Sigma-Aldrich, St Louis, MO).

### Quantitative RT-PCR (qRT-PCR) analysis

Total RNA was isolated from lung using the TRIzol reagent (Invitrogen, Life Technologies, Carlsbad, CA). Complementary DNA (cDNA) was synthesized from 2 μg of RNA using the Superscript III reverse transcriptase (Invitrogen). qRT-PCR was performed with the cDNA using the following-specific primers: 5’-CCA CGC TGT TTT GAC CTC CAT AG-3’ and 5’-ATT CCA CCA CTG CTC CCA TTC-3’ for A-C/EBP and 5’-ATG GAG GGG AAT ACA GCC C-3’ and 5’-TTCTTTGCAGCTCCTTCGTT-3’ for β-actin as normalization control. The qRT-PCR condition used was as follows: denaturation at 95°C for 15 sec, annealing at 60°C for 15 sec, and extension at 72°C for 30 sec for 40 cycles.

## Abbreviations

C/EBP: CCAAT/enhancer binding proteins; NNK: 4-(methylnitrosamino)-1-(3-pyridyl)-1-butanone; Dox: Doxycycline; CCSP: Clara cell secretory protein.

## Competing interests

All authors declare that they do not have any competing interests.

## Authors’ contribution

SK designed the study, partiscipated in carcinogenesis experiments, analyzed the data, preparing the figures and tables, and wrote a paper. JP and TK carried out carcinogenesis experiments, and prepared histological samples. MY prepared histological samples and performed immunohistochemistry. CV participated in designing carcinogenesis experiments, data analysis and interpretation of the results. JMW participated in designing carcinogenesis study, examined histological specimens, and analyzed the data and helped preparing the tables. All authors read and approved the final manuscript.

## Authors’ information

Current address for TK: Laboratory of Cell Growth and Differentiation, Institute of Molecular and Cellular Biosciences, The University of Tokyo, Tokyo 113–0032, Japan
